# Pulmonary Alveolar Proteinosis and Multiple Infectious Diseases in a Child with Autosomal Recessive Complete IRF8 Deficiency

**DOI:** 10.1007/s10875-022-01250-4

**Published:** 2022-03-26

**Authors:** Jérémie Rosain, Andrea Bernasconi, Emma Prieto, Lucia Caputi, Tom Le Voyer, Guadalupe Buda, Marcelo Marti, Jonathan Bohlen, Anna-Lena Neehus, Claudio Castaños, Rosario Gallagher, Karim Dorgham, Matias Oleastro, Laura Perez, Silvia Danielian, Jose Edgardo Dipierri, Jean-Laurent Casanova, Jacinta Bustamante, Mariana Villa

**Affiliations:** 1 Laboratory of Human Genetics of Infectious Diseases, Necker Branch, INSERM U1163, Necker Hospital for Sick Children, 24 Boulevard du Montparnasse, Paris, France; 2 Paris Cité University, Paris, France; 3 Department of Immunology and Rheumatology, “J. P.Garrahan” National Hospital of Pediatrics, Buenos Aires, Argentina; 4 Department of Biological Chemistry, School of Natural and Exact Sciences, University of Buenos Aires, Buenos Aires, Argentina; 5 Bitgenia, Buenos Aires, Argentina; 6 Institute of Biological Chemistry, School of Natural and Exact Sciences, IQUIBICEN, University of Buenos Aires, CONICET, Buenos Aires, Argentina; 7 Department of Pulmonology, “J. P.Garrahan” National Hospital of Pediatrics, Buenos Aires, Argentina; 8 Intensive Care Unit, “J. P.Garrahan” National Hospital of Pediatrics, Buenos Aires, Argentina; 9 Centre for Immunology and Microbial Infections, CIMI-Paris, Sorbonne University, INSERM, Paris, France; 10 Unit of Medical Genetics, Mother and Children Hospital Dr Hector Quintana, San Salvador de Jujuy, Jujuy, Argentina; 11 St. Giles Laboratory of Human Genetics of Infectious Diseases, Rockefeller Branch, The Rockefeller University, New York, NY USA; 12 Howard Hughes Medical Institute, New York, NY USA; 13 Department of Pediatrics, Necker Hospital for Sick Children, AP-HP, Paris, France; 14 Study Center for Primary Immunodeficiencies, Necker Hospital for Sick Children, AP-HP, Paris, France

**Keywords:** Pulmonary alveolar proteinosis, BCG, Cerebral calcifications, Myeloid cells, IRF8

## Abstract

**Background:**

Autosomal recessive (AR) complete IRF8 deficiency is a rare severe inborn error of immunity underlying an absence of blood myeloid mononuclear cells, intracerebral calcifications, and multiple infections. Only three unrelated patients have been reported.

**Materials and Methods:**

We studied an Argentinian child with multiple infectious diseases and severe pulmonary alveolar proteinosis (PAP). We performed whole-exome sequencing (WES) and characterized his condition by genetic, immunological, and clinical means.

**Results:**

The patient was born and lived in Argentina. He had a history of viral pulmonary diseases, disseminated disease due to bacillus Calmette-Guérin (BCG), PAP, and cerebral calcifications. He died at the age of 10 months from refractory PAP. WES identified two compound heterozygous variants in *IRF8*: c.55del and p.R111*. In an overexpression system, the p.R111* cDNA was loss-of-expression, whereas the c.55del cDNA yielded a protein with a slightly lower molecular weight than the wild-type protein. The mutagenesis of methionine residues downstream from c.55del revealed a re-initiation of translation. However, both variants were loss-of-function in a luciferase assay, suggesting that the patient had AR complete IRF8 deficiency. The patient had no blood monocytes or dendritic cells, associated with neutrophilia, and normal counts of NK and other lymphoid cell subsets.

**Conclusion:**

We describe the fourth patient with AR complete IRF8 deficiency. This diagnosis should be considered in children with PAP, which is probably due to the defective development or function of alveolar macrophages.

**Supplementary Information:**

The online version contains supplementary material available at 10.1007/s10875-022-01250-4.

## Introduction

Interferon regulatory factor 8 (IRF8) is a transcription factor that controls the development and function of mammalian myeloid hematopoietic cells [1–6], but also their function, including their response to interferon gamma (IFN-γ) [1, 7]. Autosomal recessive (AR) complete IRF8 deficiency is a severe inborn error of immunity (IEI) that has been reported in three unrelated patients [8–11], originating from Ireland [8, 9], England [10], and China [11]. The three patients presented multiple infections [8–11], including oral candidiasis [8, 9], respiratory infections caused by rhinovirus [8, 10], influenza virus [10], or mycoplasma [10], and adverse reactions to live BCG [8, 9] and measles, mumps, and rubella (MMR) vaccines [10]. Two of the patients underwent brain imaging, which revealed intracerebral calcifications [8, 10]. The myeloid compartment was severely impaired in all three patients, with peripheral leukocytosis caused by massive neutrophilia (up to 50 to 100 G/L) [8–11], and a lack of blood mononuclear myeloid cells, including both monocytes [8–11] and dendritic cells (DCs) [8–10]. One of these patients died [11], whereas the other two were successfully treated by hematopoietic stem cell transplantation (HSCT), one at the age of nine months [8, 9] and the other at the age of four years [10]. Four different private variants underlying AR complete IRF8 deficiency have been described: three missense variants (p.R83C [10], p.K108E [8, 9], and p.R291Q [10]) experimentally proven to be loss-of-function (LOF) in an overexpression system, and a truncating variant (p.R111* [11]) not functionally tested. Another patient with NK cell abnormalities has been described and was found to be compound heterozygous for two missense variants (p.A201V and p.P224L) that were, nevertheless, neutral in an overexpression system [12]. Thus, AR complete IRF8 deficiency has been reported in only three unrelated patients to date. We describe here a fourth patient with AR complete IRF8 deficiency. This patient differed from the others in presenting a pulmonary alveolar proteinosis (PAP) phenotype.

## Results

### A Patient with Severe Viral and Mycobacterial Diseases, PAP, and Cerebral Calcifications

**Fig. 1:**
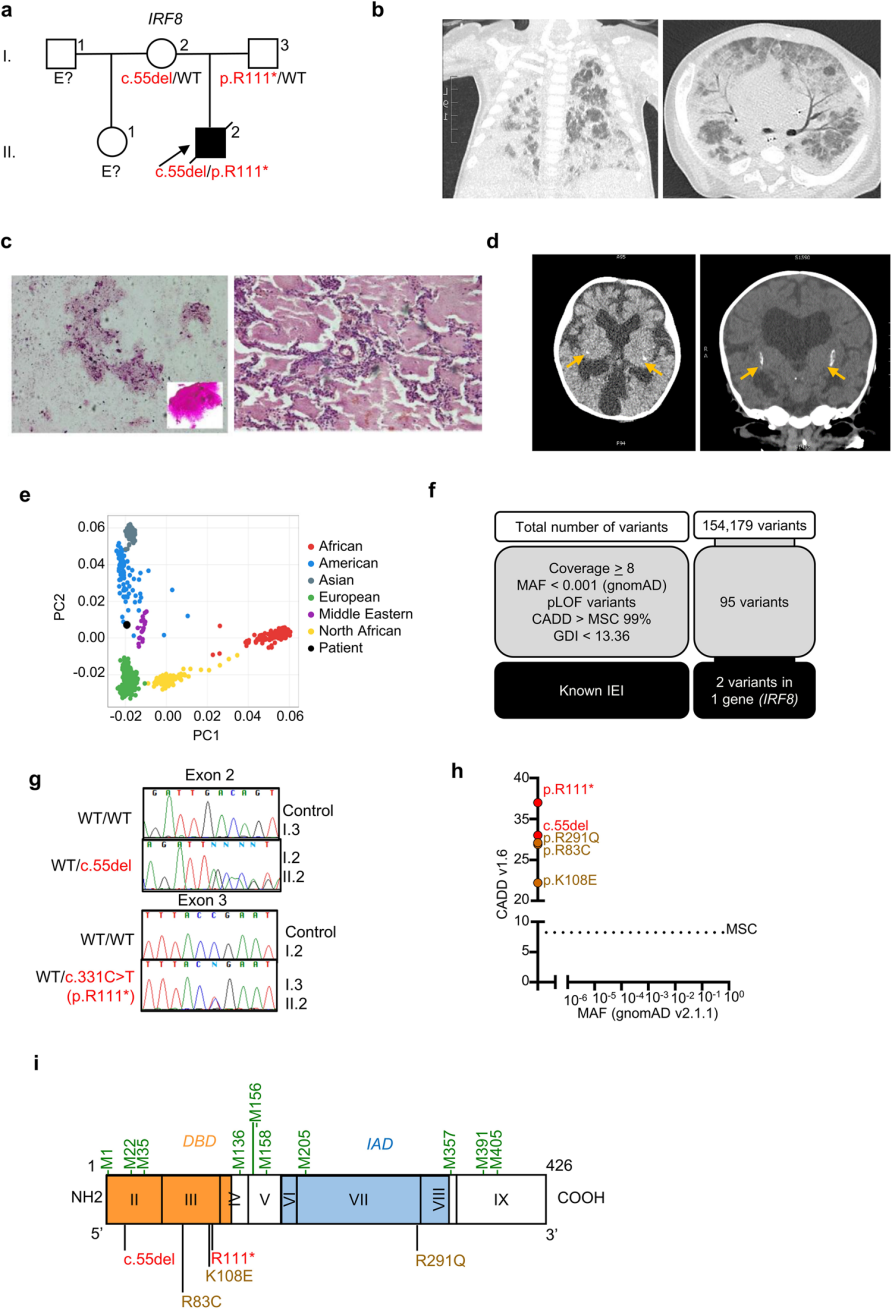
Genetic and clinical features in a patient with autosomal recessive IRF8 deficiency. **a** Pedigree of the Argentinian family. Generations are indicated by Roman numerals (I–II), and each individual is indicated by an Arabic numeral (1–3). The patient is represented by a closed black symbol, a black diagonal line (deceased), and an arrow. “E?” indicates individuals of unknown genotype. **b** Computed tomography (CT) scan of the thorax. **c** Pathology examinations of brochoalveolar lavage (BAL) and lung biopsy specimen. **d** Brain CT showing cerebral calcification (arrows). **e** Principal component analysis (PCA) showing the origins of the patient plotted on the main ethnic origins extracted from the 1000 Genomes database and our own WES database. **f** Analyses of WES data and identification of two heterozygous coding variants in *IRF8*. **g** Electropherogram of exons 2 and 3 showing the variants (c.55del and p.R111*) found in the patient (II.2) relative to healthy controls and his parents (I.2 and I.3). **h** Minor allele frequency (MAF) and combined annotation-dependent depletion (CADD) score of the heterozygous *IRF8* variants found in the patient (red symbols) and of all variants found in patients with AR complete IRF8 deficiency (brown symbols). The dotted line corresponds to the mutation significance cutoff (MSC), with its 99% confidence interval. **i** Diagram of the human IRF8 protein, showing the DNA-binding domain (DBD) and IRF-associated domain 1 (IAD). Coding exons are indicated by Roman numerals (II to IX). The new reported variants are indicated in red, and the variants previously identified as responsible for AR complete IRF8 deficiency are shown in brown. All methionine residues are indicated in green

The patient (II. 2) was a boy born in 2019 to non-consanguineous parents originating from and living in Jujuy, in the North of Argentina (Fig. 1a). His maternal half-sister and his two parents are healthy. He was born at 36 weeks of gestation, with a normal weight and length. Ventriculomegaly was diagnosed on prenatal ultrasound. The patient received BCG and hepatitis B vaccines at birth. He was admitted to the intensive care unit (ICU) at the age of one month, for sepsis and respiratory distress requiring assisted mechanical ventilation. No microorganisms were documented during this first episode, and the patient improved, thanks to empirical broad-spectrum antibiotics. At 4 months of age, he was hospitalized for a second episode of respiratory distress syndrome. Multiplex PCR on nasopharyngeal secretions was positive for a non-SARS-CoV-2 coronavirus but negative for adenovirus, influenza virus, metapneumovirus, *Mycoplasma pneumoniae*, and *Pneumocystis jirovecii*. PCR tests on blood for Epstein-Barr virus (EBV), cytomegalovirus (CMV), human herpesvirus 6 (HHV6), and HIV yielded negative results. *Acinetobacter baumanii* was cultured from bronchoalveolar lavage (BAL). The patient presented one episode of febrile convulsion. A cerebrospinal fluid (CSF) sample collected at the time was found to contain no cells, and normal protein and glucose levels. PCR on CSF for enterovirus, EBV, varicella-zoster virus (VZV), CMV, herpes simplex virus (HSV), and HHV6 yielded negative results. No mycobacterial culture was performed on CSF. The patient was treated with meropenem, vancomycin, colistin, trimethoprim/sulfamethoxazole, and liposomal amphotericin. At the age of 7 months, an axillary adenopathy, 2 × 3 cm in size, was detected at the BCG vaccination site. *M. bovis*-BCG was isolated from BAL, and the patient was treated with pyrazinamide, ethambutol, rifampicin, and levofloxacin.

A computed tomography (CT) scan of the thorax found mediastinal structures without density alterations, with a thickening of the septal interstitium, overlapping ground glass opacities with a cobblestone pattern and a tendency to consolidate at the base of the lungs (Fig. 1b). These findings were consistent with PAP, which was confirmed by pathological examinations of BAL and of lung biopsy (Fig. 1c). The patient was treated with a bolus of steroids and partial lung washes that were performed weekly at the ICU, with a good response at the start of treatment. The patient also presented facial dysmorphism, with a very wide fontanel, mid facial hypoplasia, bilateral keratoconus, a short nose and neck, retrognathia, and short stature (− 3 SD at the age of one month). Brain CT scan was performed at the age of seven months and showed ventriculomegaly with mega-cisterna magna, cerebellar hypoplasia, mesencephalic, temporal, ventricular, and ependymal calcifications (Fig. 1d). The patient died at the age of 10 months whilst in palliative care for respiratory failure. Overall, he presented susceptibility to life-threatening mycobacterial (BCG-osis) and viral (coronavirus) infections, associated with severe PAP, and cerebral calcifications.

### Identification of Compound Heterozygous IRF8 Variants

Whole-exome sequencing (WES) was performed for this patient. The homozygosity rate inferred from WES was low (0.37%), consistent with the absence of consanguinity between the two parents. Principal component analysis (PCA) confirmed that the patient was of Latino American descent (Fig. 1e). An analysis of rare variants with a minor allele frequency (MAF) < 10^−3^, predicted to be deleterious, for genes with a low gene damage index, identified 95 variants (Fig. 1f). Only two of these variants concerned a gene known to be involved in IEI, *IRF8*. One of these heterozygous variants was a single-base pair deletion (c.55del) predicted to create a frameshift (p.D19Tfs*8), and the other was a single-base pair substitution (c.331C > T) that was predicted to result in a nonsense variant (p.R111*) (Supplementary Fig. 1a). Both variants were confirmed by Sanger sequencing (Fig. 1g). The c.55del variant was inherited from the patient’s mother, whereas the c.331C > T variant was inherited from the patient’s father; the patient was therefore compound heterozygous (Fig. 1a and g). Both variants were predicted to be deleterious, with combined annotation-dependent depletion (CADD) scores of 33 for c.55del and 37 for p.R111*. These values are above the 99% mutation significance cutoff (MSC) of 8.2 for *IRF8* [2] (Fig. 1h). The p.R111* variant was previously reported in a Chinese patient with AR IRF8 deficiency [11]. However, neither of these variants has ever been reported in database of germline variants such as the public databases ExAC, gnomAD v2.1.1 or v3.1.1 [4], BRAVO/TOPmed freeze 8 [5], and ATAV [6] containing WES or whole-genome sequencing (WGS) data for more than 150,000 individuals, or in the WES data of our in-house cohort of more than 15,000 patients with severe infectious diseases (Fig. 1h). We also screened for copy number variants (CNV), in particular for homozygous or hemizygous large deletions [13]. Using WES data, targeted screening for heterozygous large deletions encompassing *TBX1* locus was negative. No CGH or SNP microarray was performed due to the lack of material. No relevant rare or private variants in other genes for which mutations are known to be associated with PAP [14–16] (namely, *ADA*, *ABCA3*, *CSF2RA*, *CSF2RB*, *GATA2*, *MARS*, *NKX21*, *RAB5B*, *SFTPB*, *SFTPC*, and *SLC7A7*), or susceptibility to mycobacterial disease [17] were identified by WES. However, biallelic mutations of *IRF8* are known to underlie syndromic MSMD [1, 8–10]. These results suggest that this patient had AR IRF8 deficiency.

### The IRF8 Variants of the Patient Are Loss-of-Function

**Fig. 2:**
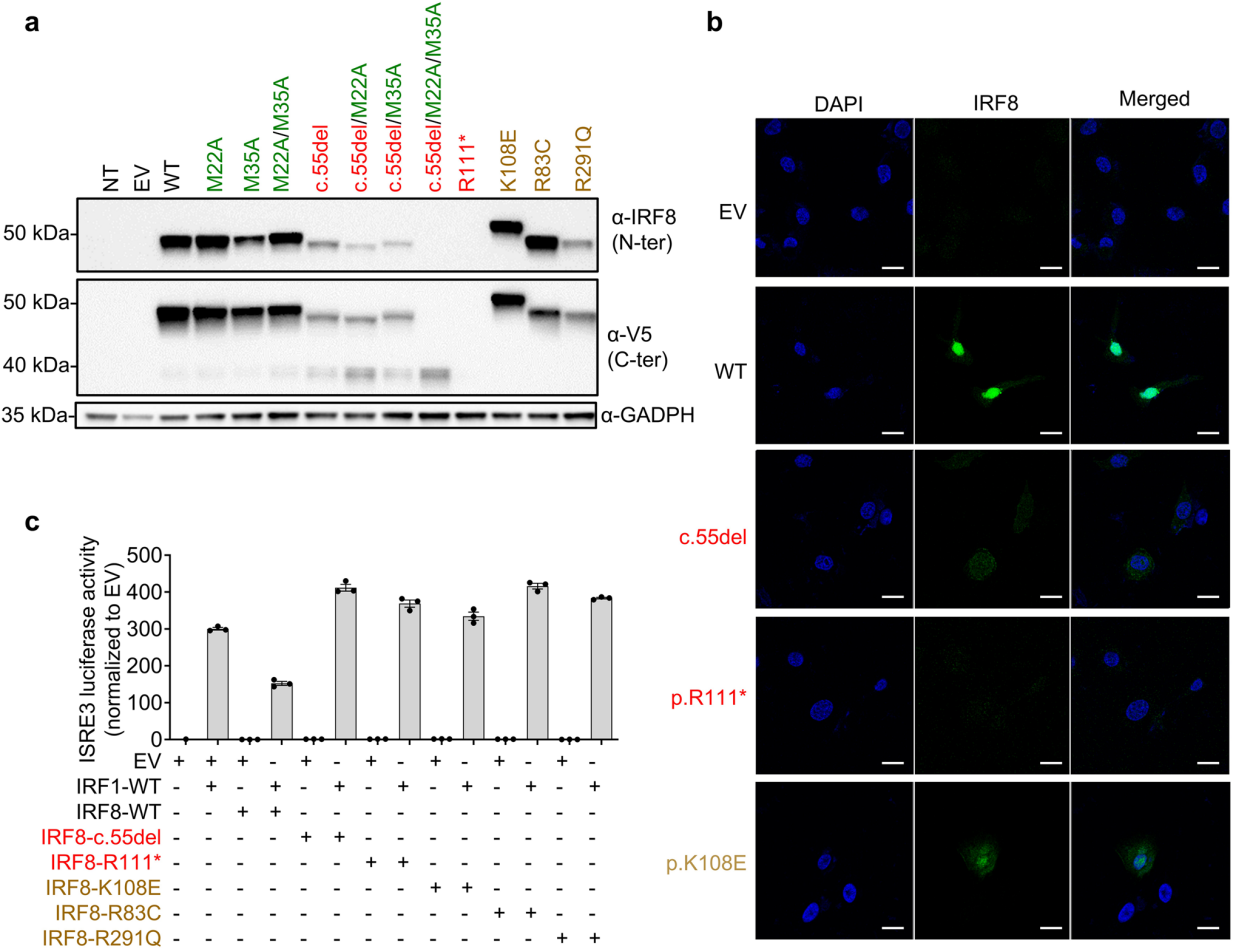
Expression and function of IRF8 variants in an overexpression system. **a** Western blots performed with total protein extracts from HEK293T cells either non-transfected (NT) or transfected with empty plasmid (EV), wild-type (WT), or *IRF8* variants (of the variants of the patient) are shown in red; the methionine (M) residues replaced by alanines (A) are shown in green, and the variants identified in previously reported IRF8-deficient patients are shown in brown. IRF8 was detected with a monoclonal antibody directed against the N-terminus of IRF8 or an antibody directed against the C-ter V5 tag. An antibody against GAPDH was used as a loading control. Data are representative of n = 2 independent experiments. **b** Confocal microscopy of HeLa cells transiently transfected with an EV, WT, or mutants IRF8, stained with DAPI and an antibody directed against the N-terminus of IRF8. The scale bar represents 20 µm. **c** Dual-luciferase ISRE3 reporter activity of HEK293T cells transfected with EV or with various *IRF8* cDNAs. Bars represent the mean and standard deviation (SD) of triplicates. Results are representative of two independent experiment

Both variants of the patient were located in the DNA-binding domain of the IRF8 protein (Fig. 1i). We studied their impact by transiently transfecting human embryonic kidney (HEK)293 T cells with the wild-type *IRF8* (WT; NM_002198. 2), or mutant c.55del or p.R111* cDNAs, and with the three previously described LOF mutants as controls (p.K108E [8, 9], p.R83C [10], p.R291Q [10]). Constructs were epitope-tagged at the carboxy-terminus (V5). Transfection was efficient, as shown by qPCR, with similar levels of *IRF8* cDNA detected in HEK293T cells transfected with the WT and mutant alleles (Supplementary Fig. 1b). Immunoblotting of cell extracts with either the monoclonal IRF8 antibody recognizing p.G85 at the amino-terminus or an antibody recognizing the carboxyterminal V5 tag showed that the WT form, and the p.K108E, p.R83C, and p.R291Q variants were produced at the expected molecular weight (MW) (Fig. 2a). Surprisingly, a band was detected at a molecular weight slightly lower than expected for the c.55del variant, whereas no protein was detected at all for the p.R111* variant (Fig. 2a). Two ATG codons downstream from c.55del and upstream from p.R111* were identified and predicted in silico to lead to a re-initiation of translation (encoding p.M22 and p.M35, respectively) [11]. We tested this hypothesis, by mutating the corresponding methionine codons (p.M22 and p.M35) to create a methionine-to-alanine (ATG > GCG) substitution. In the WT-*IRF8* cDNA, the mutation of p.M22 (WT/M22A), p.M35 (WT/M35A), or both (WT/M22A/M35A) had no effect on the MW of the protein produced (Fig. 2a). Conversely, in c.55del cDNA, the mutation of p.M22 (c.55del/M22A) resulted in protein with a slightly lower MW, whereas the mutation of p.M35 (c.55del/M35A) resulted in protein of similar MW to the c.55del protein (Fig. 2a). The substitution of both methionine residues (c.55del/M22A/M35A) completely abolished protein production (Fig. 2a). Thus, in overexpression systems, the c.55del mutation leads to a re-initiation of translation at the downstream ATG encoding p.M22. The resulting protein is truncated by 21 amino acids and has a lower MW (p.M1_S21del). The c.55del variant displayed impaired accumulation in the nucleus, like the previously described p.K108E mutant [9] as shown by analysis of cytoplasmic and nuclear extracts from HEK293T cells (Supplementary Fig. 1c) and by confocal microscopy on HeLa cells (Fig. 2b). In luciferase assays on HEK293T cells, the c.55del and p.R111* proteins, like the three previously reported mutants (p.K108E [8, 9], p.R83C [10], p.R291Q [10]), were completely unable to inhibit IRF1-driven ISRE-transcriptional activity (Fig. 2c). These results indicate that both the variants from the patient are LOF and suggest that the patient displayed AR complete IRF8 deficiency.

### Impairment of Leukocyte Development and Function in the Patient

**Fig. 3:**
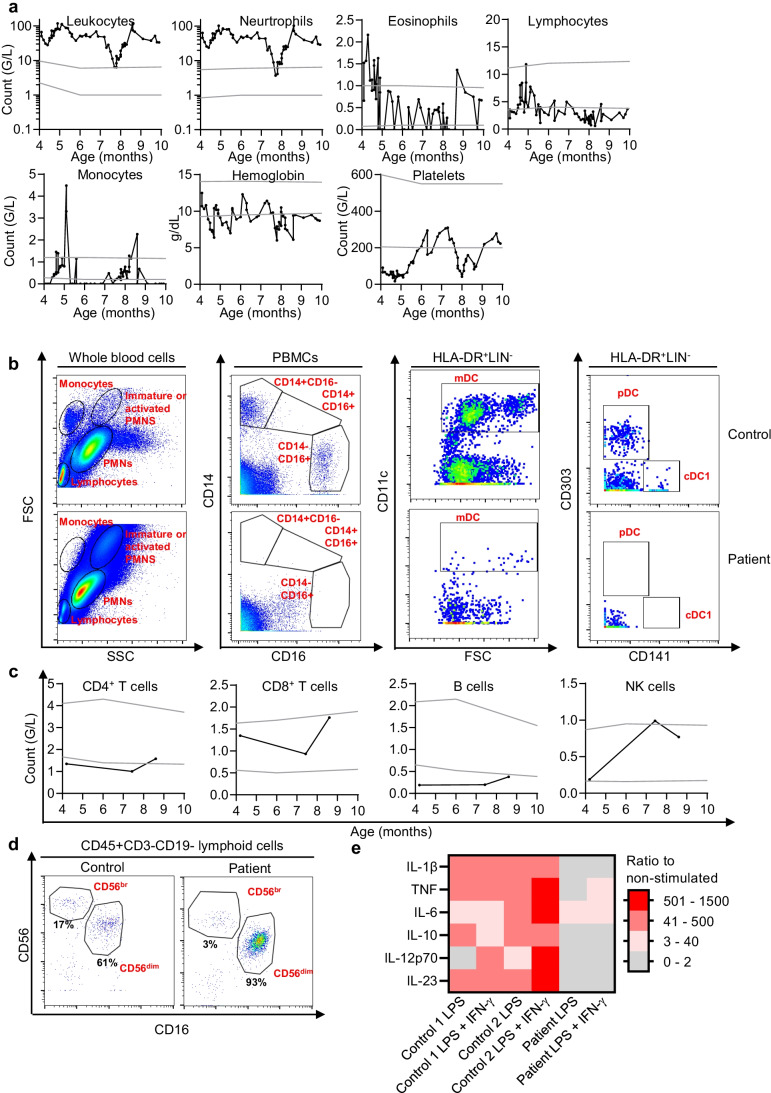
Counts and function of peripheral leukocytes, hemoglobin, and platelets. **a** Monitoring of the levels of leukocytes, neutrophils, eosinophils, lymphocytes, monocytes, hemoglobin, and platelets in the patient. **b** Flow cytometry study of monocytes, mDC, cDC1, and pDC. **c** Monitoring of the levels of CD4^+^ and CD8^+^ T cells, B cells, and NK cells in the patient (black line). **d** Dot plot of CD16^+^ and CD56^+^ expression for NK cells. **e** Induction of cytokines, measured in the supernatant of two controls, and of the patient, after stimulation with LPS ± IFN-γ. The results are expressed as a ratio relative to non-stimulated cells

The patient displayed constant marked leukocytosis due to the proliferation of polymorphonuclear neutrophils (Fig. 3a). Monocytes were not identified with any certainty on conventional hemograms (probably confused with large lymphocytes) (Fig. 3a), and the complete absence of these cells was demonstrated by flow cytometry (Fig. 3b). Bone marrow aspiration was performed, and no signs of lymphoma or leukemia were detected. The patient had slightly high plasma FTL3 ligand levels (Supplementary Fig. 1d). Circulating pDC and cDC1 were also found to be absent by flow cytometry (Fig. 3b). The number of NK cells was within the normal range (Fig. 3c), and the distribution of CD56^br^ and CD56^dim^ cells was normal (Fig. 3d). The patient displayed mild CD4^+^ T lymphopenia (Fig. 3c), with a normal percentage of naïve CD4^+^ T cells (Table 1). T_H_17, T_H_1, and Treg CD4^+^ T cells were present at their usual percentages, whereas the percentage of T_H_F cells was slightly lower than normal (Table 1). CD8^+^ T cells were present within the normal range (Fig. 3c). The patient presented B-cell lymphopenia (Fig. 3c) and had low levels of memory B-cell subsets (CD27^+^, CD27^+^IgD^+^, and CD27^+^IgD^−^) for his age (Table 1). Platelet levels were either low or in the low end of the normal range (Fig. 3a). Despite an absence of pDCs, plasma IFN-α levels were at upper range of controls in the patient (Supplementary Fig. 1d). Following stimulation with IL-12, phytohemagglutinin (PHA)-activated peripheral blood mononuclear cells (PBMCs) or PHA-blasts from the patient displayed normal levels of intracellular IFN-γ production by CD8^+^ and CD8^−^ T cells (Supplementary Fig. 1e). Whole-blood activation with LPS + IL-12 resulted in an impaired IFN-γ production (data not shown). Consistent with a complete absence of circulating myeloid mononuclear cells, following activation with LPS and IFN-γ, whole blood from the patient was completely unable to produce IL-1β, IL-10, IL-12p70, and IL-23, and TNF levels were low (Fig. 1e). Screening for anti-GM-CSF and anti-type I IFN autoantibodies, which can underlie PAP and severe COVID-19 pneumonia [18], respectively, was negative in a plasma sample of the patient collected at the age of nine months (Supplementary Fig. 1f and 1g).

**Table 1:** Immunophenotyping of various immune cell subsets in peripheral blood samples from the patient

		4 months	7 months	9 months	Normal range
CD4^+^ T-cell subsets	CD45RA^+^ (% CD4^+^ T cells)	87	69	nd	65.0–80.6
	CD45RO^+^ (% CD4^+^ T cells)	17	nd	nd	9.7–20.6
	T_H_17 (% CD4^+^ T cells)	nd	nd	0.49	0.25–1.0
	T_H_1 (% CD4^+^ T cells)	nd	nd	2.4	2.0–4
	Treg (% CD4^+^ T cells)	nd	nd	14	6.10–9.9
	T_H_F (% CD4^+^ T cells)	nd	nd	3.9	7.8–8.6
B-cell subsets	CD27^+^ (% of CD19^+^ cells)	nd	nd	11	12.74–24.20
	CD27^+^IgD^−^ (% of CD19^+^ cells)	nd	nd	5	1.97–10.47
	CD27^+^IgD^+^ (% of CD19^+^ cells)	nd	nd	6	8.38–14.82
NK-cell subsets	NK^dim^ (% of NK cells)	nd	nd	93	
	NK^bright^ (% of NK cells)	nd	nd	3	

*nd* not determined

## Discussion

The newly identified patient with AR complete IRF8 deficiency described here presented clinical and immunological features common to the other three patients reported [8–11]: susceptibility to severe infectious disease (including BCG-osis following exposure to BCG vaccine and viral infections), intracerebral calcifications [8–10], and absence of circulating monocytes [8–11] and DCs [8–10], with massive neutrophilia [8–11]. FTL3 ligand levels were slightly elevated, as previously described in another patient with AR complete IRF8 deficiency [8]. This is seen in patients with bone marrow failure [19, 20]. The other three patients described had normal [11] or increased [8–10] NK cell counts, while the patient reported here had a normal count of NK cells, and a normal distribution of CD56^dim^ and CD56^br^ NK cells. IRF8 is expressed by microglial cells [1], which has led to the suggestion that its complete deficiency is a microgliopathy [21, 22] resembling other conditions caused by mutations of genes expressed by microglia, variants of which have been shown to lead to intracerebral calcification [21, 22]. Follow-up of patients with AR complete IRF8 deficiency, including patients undergoing HCST, would be required to determine whether the cerebral calcifications observed underlie any neurological phenotype. In addition to intracerebral calcifications, the patient described here also had other brain lesions and signs of developmental features (including mid facial hypoplasia and keratoconus) that had not been described in other patients with AR complete IRF8 deficiency. Heterozygous CNVs or complex rearrangements were not ruled out by GCH array due to lack of material. A description of more patients is needed to conclude if these features are related to AR complete IRF8 deficiency. The patient reported here also presented severe refractory PAP, as demonstrated by pathological examinations of BAL and a lung biopsy. PAP was not reported in any of the other three patients with AR complete IRF8 deficiency, despite respiratory distress [8–11] and in-depth respiratory evaluations in one of these patients [10], including BAL [10]. PAP is mostly caused by inborn errors of surfactant homeostasis or the GM-CSF pathway in children, or by autoantibodies against GM-CSF in adults [14]. Interestingly, PAP has been reported in patients with autosomal dominant GATA2 deficiency who also display impaired monocytes and DC counts [20, 23, 24]. The impaired development or function of intra-alveolar macrophages is probably the cellular basis of PAP in these patients.

## Materials and Methods

### DNA Extraction, WES, and Sanger Sequencing

Genomic DNA was extracted from whole-blood samples with the QIAamp DNA Mini Kit (QIAGEN). Exons were captured with the Agilent SureSelect Human All Exon V5 kit and sequenced on an Illumina platform with a read length of 100 base pairs and a main depth coverage of 100 × . Paired-end reads were mapped onto the human reference genome GRCh37 with the Burrows-Wheeler aligner (BWA), and the following analysis was performed as recommended by the Broad Institute guidelines, with the Genome Analysis Toolkit (GATK). Duplicate reads were marked with PICARD, and variants were called with “Haplotype Caller” from GATK (100,341 variants were obtained). We annotated the final VCF file with SnpEff/SnpSift. The following databases were included: dbSNP, ExAC, 1000 Genomes, ClinVar, and the prediction tools Polyphen2, SIFT, and Mutation Taster. Variant prioritization was performed with the web-based server B_Platform (Bitgenia, www. bitgenia. com). Candidate variants were analyzed for presence in the set of candidate genes, population frequency, possible impact on the protein sequence, and previous clinical reports in databases.

Exons 2 and 3 of *IRF8* were amplified from genomic DNA with the primers 5′-GCCAGCACCTTTGCTGCAAACCTC-3′ and 5′-GATGTCACTCAACATCTCCACT-3′, and 5′-GATGTGTGGGGTCCTGGTCATGGTG-3′ and 5′-ACTATGTGCCCCAGACCCTGTGTCT-3′ primers, respectively, at a Tm of 60 °C, with GoTaq DNA Polymerase (#M3005, Promega). They were then sequenced by the Sanger sequencing method with Big Dye Terminator v3. 1 (Thermo Fisher Scientific), and subjected to capillary electrophoresis (#A30469, Applied Biosystems 3500xL system, Thermo Fisher Scientific).

### Plasmid Cloning and Mutagenesis

Empty vector or plasmid (EV) pcDNA3.1 was obtained from Thermo Fisher Scientific, and pCMV6 EV and *IRF1*-WT-DDK tagged plasmid (#RCPS100001 and #RC203500, respectively) were obtained from Origene. *IRF8*-WT-V5-tagged (NM_002163) was inserted not the vector by cloning. Constructs carrying single-nucleotide mutant alleles were generated from this plasmid by mutagenesis with the appropriate primers, with the Pfu Ultra II Fusion HS DNA (#600,674, Agilent) polymerase or CloneAmpHiFi PCR Premix (#639,298, Takara) followed by digestion with *Dpn*I (#R0176L, New England Biolab). Plasmids were amplified in competent cells of *E. coli* (#C3019H, New England Biolab) and were purified with a maxiprep kit (#12,663, Qiagen). HEK293T cells were transiently transfected with the various constructs at a concentration of 2.5 µg/mL, in the presence of Lipofectamine LTX reagent with PLUS (#15,338,100, Thermo Fisher Scientific) in accordance with the manufacturer’s instructions.

### Western Blotting

Total protein extracts were prepared by mixing cells with modified radioimmunoprecipitation assay buffer supplemented with protease inhibitors (Complete, Roche) and phosphatase inhibitor cocktail (PhosphoStop, Roche), 0.1 mM dithiothreitol (DTT; Life Technologies), 10^−3^ mM Na_3_VO_4_, and 1 mM PMSF, and incubating for 40 min on ice. The cytoplasmic and nuclear contents of the cells were separated with NE-PER nuclear and cytoplasmic extraction reagents (#78,835, Thermo Fisher Scientific). Equal amounts of protein, according to a Bradford protein assay (#5,000,002, Biorad), were resolved by SDS-PAGE in a Criterion TGX 10% precast gel (#5,671,033, #5,671,034, or #5,671,035, Biorad), and the resulting bands were transferred to a nitrocellulose membrane (#1,704,159, Biorad). Membranes were probed with antibodies directed against IRF8 (unconjugated, clone D20D8, #5628, Cell Signaling), the V5 tag (HRP-coupled, Thermo Fisher Scientific), lamin A/C (HRP-conjugated, clone E-1, #sc-376248, Santa Cruz), or GADPH (unconjugated, clone D16H11, #5174, Cell Signaling). Unconjugated antibodies were detected by incubation with HRP-conjugated goat anti-rabbit IgG (H + L) antibodies (#1,706,515, Biorad). Binding was detected by incubation with the Clarity Western ECL substrate (Biorad, #1,705,061) or SuperSignal West Femto (Thermo Fisher Scientific, #34,096) with ChemiDoc MP (Biorad). The Spectra Multicolor Broad-Range Protein Ladder (#26,623, Thermo Fisher Scientific) or the Chameleon Duo Prestained Protein Ladder (#928–6000, Licor) was used to provide molecular weight markers. Membranes were stripped with Restore Western Blot Stripping Buffer (#21,063, Thermo Fisher Scientific). Images were analyzed with Imagine Lab 6. 0. 1 build 34 (Bio-Rad Laboratories).

### Luciferase Assay

We used the ISRE3 reporter plasmid (pGL4. 10[luc2] backbone, Promega #E6651), as previously described [25]. HEK293T cells in 96-well plates were transiently transfected with the (ISRE) reporter plasmid (100 ng/well and 100 µL of DMEM-10% FBS medium) and the pRL-SV40 plasmid (Promega, #E2231, 40 ng/well), with or without the *IRF1* WT p.CMV6 plasmid (50 ng/well), with or without *IRF8*-WT or mutant forms, in the presence of the Lipofectamine LTX kit (Thermo Fisher Scientific, #15,338–100) and with or without EV, to ensure that the same amount of plasmid was present in each well, in accordance with the manufacturer’s instructions. Cells were used for the ISRE assay with the Dual-Luciferase system kit (Promega #E1980), according to the manufacturer’s protocol, 24 h after transfection. Signal intensity was determined with a Victor X4 plate reader (Perkin Elmer). Experiments were performed in triplicate, and results are expressed as dual reporter activity.

### Confocal Microscopy

HeLa cells were plated on chambered coverslip (#80,826, iBidi) and were left untransfected or were transiently transfected with *IRF8*-WT or mutant cDNA constructs or with EV. Twenty-four hours later, cells were fixed 15 min in 4% formaldehyde in phosphate-buffered saline (PBS), pH 7.4 at 37 °C. The cells were then incubated overnight at 4 °C with primary antibody (unconjugated IRF8, clone D20D8, #5628, Cell Signaling). They were washed three times in PBS, stained by incubation with secondary antibodies for one hour at room temperature (goat anti-rabbit IgG Alexa Fluor 488 (#A-11034), and left in ProLong Gold with DAPI (#P36931, Thermo Fisher). Cells were then visualized by confocal microscopy (× 63 oil immersion lens, SP8 gSTED, Leica). Images were analyzed using Fiji software.

### Flow Cytometry Studies

Phenotypic analyses were performed on PBMCs with standard flow cytometry (FC) methods [26], on an eight-color FACS Canto II instrument (Becton Dickinson, San Jose, CA, USA) equipped with FACSDiva software (BD). Monoclonal antibodies (mAbs) — directed against CD3, CD4, CD11B, CD14, CD16, CD19, CD20, CD21, CD24, CD25, CD27, CD38, CD39, CD45, CD45RA, CD56, CD127, CD185 (CXCR5), IgD, IFNγ, and IL-17 — were purchased from Becton Dickinson (BD), San Jose, CA, USA. Antibodies against CD1c, CD141, CD303, CD304, and CD45v500 were acquired from Miltenyi Biotec, Bergisch Gladbach, Germany. The CD13 and CD11b staining patterns of peripheral mature neutrophils were analyzed, and monocyte subsets were identified on the basis of their expression of CD14 and CD16. DCs were identified on the basis of MHC class II (HLA-DR) expression by lineage negative (LIN −) cells (DR + LIN −) and were counted. The LIN population was defined by markers specific for the T-cell (CD3^+^), B-cell (CD19^+^, CD20^+^), NK-cell (CD56^+^), and monocyte (CD14^+^) lineages. DC subsets were identified on the basis of blood DC antigens (BDCA) 1–4 in the DR^+^ LIN^−^ population: CD1c (BDCA-1) and CD141 (BDCA-3), which were used to recognize cDCs, and CD303 (BDCA-2) and CD304 (BDCA-4), which were used to identify pDCs. DC percentages are expressed relative to total CD45^+^ leukocytes. Treg cells were identified as a CD127^dim^CD25^high^ population on CD3^+^CD4^+^ cells. For the quantification of T_H_17 and T_H_1 cells, two million PBMCs were left unstimulated or were stimulated with phorbol 12-myristate 13-acetate (PMA, 100 ng/mL) and ionomicin (1 µg/mL) (Sigma Aldrich) for 15 h at 37 °C, under an atmosphere containing 5% CO_2_, in the presence of brefeldin A (10 µg/mL). Cells were fixed and permeabilized with Cytofix/Cytoperm and Perm/Wash buffer from Becton Dickinson (BD) and stained with antibodies directed against CD3, CD4, IFN-γ, and IL-17. We analyzed the proportions of T_H_17 and T_H_1 cells among CD4^+^ T cells.

### Whole-Blood Cell Stimulation and ELISA on Supernatants and Plasma

Whole-blood samples were diluted 1/2 with RPMI 1640 (GIBCO BRL) and stimulated with lipopolysaccharide (LPS) from *Salmonella* Minnesota (100 ng/mL, Sigma-Aldrich), PMA (10^−7^ M; Sigma-Aldrich) with ionomycin (I) (10^−5^ M; Sigma-Aldrich), IL-12 (GIBCO BRL), and IFN-γ (Imukin). Patient samples and samples from two age-matched healthy controls were run in parallel. Supernatants were recovered after 48 h and were used for enzyme-linked immunosorbent assays (ELISA) for human TNF and human IFN-γ (Thermo Fischer Scientific). ELISA was performed on plasma from the patient, GATA2-deficient patients [20] and healthy donors, for human Flt-3 ligand (FLT3L) (R&D Systems, DFK00) in accordance with the kit manufacturer’s instructions. Similarly, plasma IFN-α levels were measured by SIMOA on patient, and healthy donors, in accordance with the kit manufacturer’s instructions (Quanterix). SIMOA was also performed on plasma from the patient, and healthy donors, in accordance with the kit manufacturer’s instructions. The production of myeloid cytokines was assessed after 24 h of stimulation with LPS, with or without IFN-γ, with LEGENDplex™ Human Inflammation Panel 1 (#740,809, BioLegend).

### Study of IFN-γ Induction in PHA-Blasts

PHA blasts from the patient and an age matched control were generated stimulating 1 × 10^6^ PBMC with PHA (Gibco, diluted 1/50 in RPMI 20% ABS Penicillin/Streptomycin/Glutamine) plus recombinant IL2 (Roche, 50 IU) during 48 h. After two washes with RPMI, mediumcells were divided in two aliquots and incubated either with RPMI 2% FCS (basal tube) or 0.1 µg/ml recombinant IL12 R&D (stimulated tube) for another 24 h. Then, both basal and stimulated tubes were re-stimulated with PMA 100 ng/ml and ionomycin (1 µg/ml) in RPMI 20% ABS P/S/G for 5 h in the presence of 10 µg/ml Brefeldin A (Sigma). Cells from basal and simulated tubes were then washed twice, lysed with Becton Dickinson (BD) FACS lysing, and permeabilized with BD FACS permeabilizing solution 2. Permeabilized cells were stained with IFN-γ FITC, CD3 PerCP, and CD8 PE (BD) for 30 min; washed twice with PBS /BSA; and acquired in a FACS Canto II. Percentages of IFN-γ positive cells from basal and IL12 stimulated blast cells were analyzed on gated CD3 + CD8 + and CD3 + CD8- populations on FlowJo.

### Screening of Autoantibodies to GM-CSF and Type I IFNs

Recombinant E. coli-derived human (rh)GM-CSF (#215-GMP, R&D Systems) was first biotinylated with EZ-Link Sulfo-NHS-LC-Biotin (#A39257, Thermo Fisher Scientific), according to the manufacturer’s instructions, with a biotin-to-protein molar ratio of 1:12. The detection reagent contained a secondary antibody (Alexa Fluor 647 goat anti-human IgG (#A21445, Thermo Fisher Scientific)) diluted in Rexxip F (#P0004825, Gyros Protein Technologies; 1:500 dilution of the 2 mg/ml stock to yield a final concentration of 4 μg/ml). Buffer phosphate-buffered saline, 0.01% Tween 20 (PBS-T), and Gyros Wash buffer (#P0020087, Gyros Protein Technologies) were prepared according to the manufacturer’s instructions. Plasma or serum samples were then diluted 1:100 in 0.01% PBS-T and tested with the Bioaffy 1000 CD (#P0004253, Gyros Protein Technologies) and the Gyrolab xPand (#P0020520, Gyros Protein Technologies). Cleaning cycles were performed in 20% ethanol.

Screening of autoantibodies to type I IFN-α2a 100 pg/mL, IFN-β 10 ng/mL, and IFN-ω 100 pg/mL in plasma diluted 1 to 10 were performed as previously described using a dual interferon stimulated response element (ISRE) luciferase system in HEK293T cells [18]. Results were normalized to non-stimulated condition.

## Supplementary Information

Below is the link to the electronic supplementary material.

Supplementary Figure 1 - (a) IGV viewer presentation of the IRF8 region of the genome containing the variants of the patient (c.55del and c.331C>T), in the sense orientation. (b) RT-qPCR on cDNA from HEK293T cells non-transfected (NT) or transfected with an empty plasmid (EV), wild-type (WT)-IRF8, mutated-IRF8 (red corresponds to variants found in this patient, brown corresponds to previous LOF variants identified in patients with complete IRF8 deficiency), with a probe spanning the junction between exons 2 to 3. GUSB was used for normalization. Values are expressed as means ± SEM. (c) Cytoplasmic and nuclear protein fractions extracted from HEK293T cells transiently transfected with an empty plasmid (EV), wild-type (WT)-IRF8, or constructs including the variants of the patient (c.55del and p.R111*), or variants previously identified as responsible for AR complete IRF8 deficiency (p.K108E), probed with an antibody directed against the C-ter V5 tag. An antibody against vinculin or laminin A/C was used as a loading control for the cytoplasmic and nuclear extracts, respectively. (d) Plasma FTL3 ligand level, determined by ELISA for controls, the patient, or from and a patient with GATA2 deficiency; and Plasma IFN-α2a level, determined by Simoa for controls, the patient, and patients with systemic lupus erythematosus (SLE). (e) Intracellular IFN-γ production by PHA-blast (CD8- or CD8+ T cells), assessed by flow cytometry, with and without prior stimulation with IL-12 for 24 hours. (f) Determination of GM-CSF IgG antibody levels by Gyros in plasma from healthy donors, patient, and patients with known GM-CSF antibodies. (g) Neutralizing activity toward 100 pg/mL of IFN-α2a, 100 pg/mL of IFN-ω, or IFN-β 10 ng/mL in plasma diluted 1 to 10 of healthy donors, patient, or a patient with hypomorphic biallelic RAG1 mutation. (JPG 3952 KB)

## Data Availability

All data are either included in the manuscript or available upon request.
